# The Effect of Patient Weight and Provider Training and Experience on Dosing of Rocuronium

**DOI:** 10.1155/2016/3136895

**Published:** 2016-06-26

**Authors:** N. C. Godwin, L. Rodriguez, S. Banks, B. T. Major, Y. Rodriguez

**Affiliations:** ^1^Department of Anesthesiology, Jackson Memorial Hospital/University of Miami, Miami, FL 33136, USA; ^2^Department of Health Sciences Research, Mayo Clinic, Rochester, MN 55905, USA

## Abstract

*Introduction*. Maintenance dosing of neuromuscular blocking agents is complex and varies with patient, procedure, and clinical situation. With this in mind, we sought to identify factors impacting the maintenance dosing of neuromuscular blockers as a step toward identifying best practice with respect to minimizing residual neuromuscular blockade.* Methods*. Cases utilizing rocuronium from July 1, 2010, to June 30, 2014, at the sponsoring institution were analyzed. Using a mixed model to account for repeated measures, patients were analyzed by dose and weight category as defined by the World Health Organization (eight categories ranging from very severely underweight to very severely obese) as well as by the administering provider's level of experience.* Results*. The study included 12,671 patients with a mean age of 49.7 (SD 16.7). Increasing weight category and higher levels of provider experience were associated with higher doses for rocuronium. There were no differences in initial dose or in frequency of maintenance dosing by weight category after controlling for case length.* Discussion*. The two dosing patterns identified, higher doses for overweight patients and higher doses administered by experienced providers, are modifiable factors that could enhance patient safety.

## 1. Introduction

There is ever-increasing evidence that many patients have residual neuromuscular blockade following extubation with some studies indicating an incidence as high as 60% [1, 2]. However, a large survey revealed that anesthesiologists estimate the incidence to be less than 1% [3]. Clinical implications include an increase in numerous postoperative complications including hypoxia, need for airway intervention, delayed discharge from PACU, and even perhaps mortality [4–8].

Maintenance dosing of neuromuscular blocking agents is complex and varies with patient, procedure, and clinical situation [9, 10]. There are no clearly established guidelines for maintenance dosing of neuromuscular blockade [11]. The obesity epidemic in the United States has altered the dosing practices for many medications [12]. Providers can no longer rely upon a “standard dose” of any weight-based therapy to produce a desired clinical effect. Neuromuscular blocking agents are one such group that requires providers to tailor dosing to a patient's weight [13, 14]. Furthermore, rocuronium dosing should be based on a patient's ideal body weight (IBW) rather than total body weight (TBW). This adds further complication because IBW must be calculated and is not as readily available as the patient's TBW [15].

At the sponsoring institution, which is typical of United States hospitals, most of the maintenance doses of neuromuscular blockers are administered by providers other than attending anesthesiologists. Physicians training to become anesthesiologists spend three years of residency (Clinical Anesthesia Years 1, 2, and 3; CA1, CA2, and CA3) learning the specialty under the supervision of attending anesthesiologists. While attending anesthesiologists are present at critical moments, the residents manage the case during the maintenance phase of anesthesia and give maintenance doses of neuromuscular blockade independently. Similarly, Clinical Registered Nurse Anesthetists (CRNAs) and Student Registered Nurse Anesthetists (SRNAs) administer anesthesia under the supervision of attending anesthesiologists. These providers are nurses who have completed (CRNAs) or are undergoing (SRNAs) specific training in anesthesia after nursing school who often manage the maintenance phase of anesthesia semi-independently [16]. It is not known if provider level of experience and training impacts maintenance dosing of neuromuscular blockade.

Fully eliminating residual neuromuscular blockade will likely require a multifaceted approach [17]. With this in mind, we sought to identify factors impacting the maintenance dosing of rocuronium as a step toward identifying best practice with respect to minimizing residual neuromuscular blockade.

## 2. Methods

**Figure 1:**
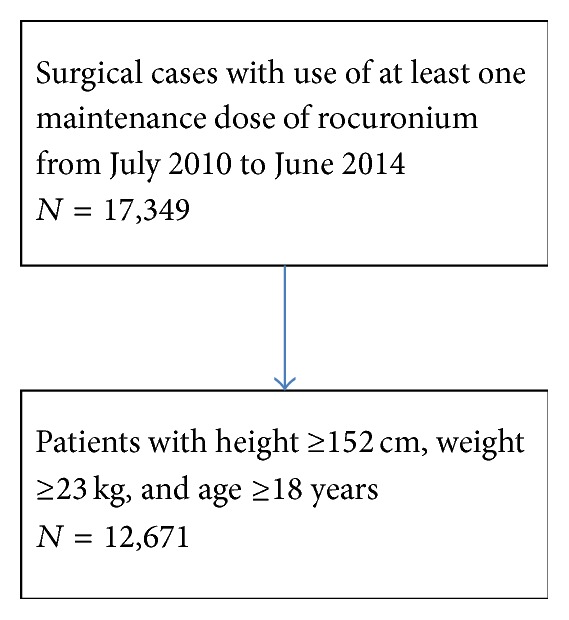
Patient inclusion criteria.

Using data from the Anesthesia Information Management System (PICIS, Version 7.1), all surgical cases utilizing neuromuscular blockers from July 1, 2010, through June 30, 2014, at the sponsoring institution were analyzed. Our database included patient demographic data such as age, height, weight, gender, and case related data such as neuromuscular blocker dose and dose date and times and anesthesia and surgery start/end date and times. Pediatric patients and cases involving patients less than five feet tall were excluded due to differences in calculating IBW for shorter patients. All patients who received at least one maintenance dose of rocuronium were included in the study (Figure 1). The dose administered in mg was converted to both mg/kg of total body weight and ideal body weight using the patient's sex, height, and weight. The recommended maximum maintenance dose was determined by assuming that no more than one-tenth of the intubating dose should be administered. Applying this rule gives a maximum maintenance dose of 0.12 mg/kg for rocuronium.

Using univariate linear mixed effects models with random effects for subject and time, the maintenance doses of rocuronium were analyzed by level of provider training (CRNA, SRNA, CA1, CA2, and CA3) and weight category as defined by the World Health Organization (eight categories ranging from very severely underweight to very severely obese). This method was chosen to account for multiple observations per subject and differences in the time between doses and time under anesthesia for each patient. The response variable in each model is maintenance dose in mg/kg of IBW. The univariate models included time, the covariate, and the interaction between time and the covariate. For all analyses a *p* value below 0.05 was considered statistically significant. All analyses were performed using SAS Version 9.3 (SAS Institute, Cary NC, USA).

## 3. Results

The study included 12,671 patients with a mean age of 49.7 (SD 16.7). 53% of patients were male, and the average height of 168.9 cm (SD 9.8) and weight of 79.8 kg (SD 18.9) resulted in a mean BMI of 27.9 (SD 6.0) and IBW of 62.6 kg (SD 8.5) (Table 1). The median procedure length was 4.8 hours (Q1, 2.9–Q3, 6.1), and maintenance doses were administered with an overall median frequency of every 92 minutes (Q1, 56 min–Q3, 158 min). Residents performed 52% of the cases and CRNAs 39% (the remaining cases were split between attending anesthesiologists, anesthesiology fellows, and SRNAs).

The overall mean maintenance dose of rocuronium was 0.29 mg/kg of IBW, which is more than twice the amount commonly recommended (0.12 mg/kg of IBW). Overall, 82% of the maintenance doses given were more than the recommended upper limits.

In the first rocuronium linear mixed effects model, increasing weight category was associated with a higher maintenance dose (overall *p* value for BMI, *p* < 0.0001). All weight categories received higher than the recommended doses, but the model demonstrated a clear pattern of patients with low weight patients receiving lower doses and heavier patients receiving higher doses. For example, Table 2 shows that patients in obese class III received 0.059 mg/kg of IBW more rocuronium per maintenance dose than patients in the normal weight category (who themselves received 0.28 mg/kg of IBW per dose, more than twice the recommended limit). There were no differences between BMI categories in dosing amounts over time.

The second rocuronium mixed effects model demonstrated different dosing patterns based on provider level of experience (overall *p* value for provider level, *p* < 0.0001). While all groups showed a consistent tendency to administer doses above the recommended dose, more inexperienced providers administered lower maintenance doses, and more experienced providers administered higher maintenance doses. For example, Table 3 shows that physician residents in their first year of training (CA1) gave 0.021 mg/kg of IBW less rocuronium per maintenance dose than CRNAs. There were no differences between different provider training and experience in dosing amounts over time.

There were too few respiratory complications (only 32 cases) documented in the anesthesia record such as residual weakness or unplanned postoperative ventilation to include in the analysis, and events occurring in the postanesthesia recovery unit after the anesthesia record was closed were not available.

## 4. Discussion

Providers administer higher maintenance doses of nondepolarizing neuromuscular blockers for obese patients, and the authors believe that this represents dosing by TBW rather than IBW. These patients often have comorbid conditions such as reduced lung compliance and obstructive sleep apnea that put them at the highest risk for the respiratory complications associated with residual neuromuscular blockade.

There are multiple factors other than obesity that warrant smaller doses of nondepolarizing doses. Frequent, careful monitoring of clinical effect by TOF and titration to the desired endpoint is essential as there is considerable individual variability. Given this complexity and the myriad reasons warranting smaller doses, it is surprising that a lower level of anesthesia training was associated with the use of lower doses. Perhaps the infrequency with which anesthesia providers recognize residual neuromuscular blockade leads over time to decreased concern for the clinical effects of deeper levels of paralysis. This is likely true for many medications, with more inexperienced providers feeling less comfortable with giving larger doses. In the case of neuromuscular blockade, especially with obese patients, this caution is warranted.

As this was a single-site, retrospective study, the generalizability of these findings is not known. No attempt was made to identify patients who might warrant the use of higher doses of neuromuscular blockade (e.g., burn patients and patients with high circulatory volume). However, given the large number of cases in the study and the relatively small portion that these patients comprise in the institution's practice, it is unlikely that removing these patients from the analysis would have altered the study results.

Routine use of twitch monitoring and other clinical signs of depth of blockade are recommended and offer guidance but are subjective and open to errors in interpretation. Maintenance dosing of neuromuscular blockade varies by provider level of training and by patient weight, and these factors likely contribute to residual neuromuscular paralysis. These data will inform future interventions and serve as a baseline from which future interventions will be evaluated in the continued effort to eliminate residual neuromuscular blockade.

## Figures and Tables

**Table 1:** Demographic, procedure, and dosing statistics.

Variable	
Age	Mean 49.7 (SD 16.7)
Gender	9,728 males (53%)
Height	Mean 168.9 cm (SD 9.8)
Weight	Mean 79.8 kg (SD 18.9)
Length of procedure	Median 4.3 hours (Q1, 2.9–Q3, 6.1)
Dosing interval	Median 92.5 minutes (Q1, 56.3–Q3, 158.4)

**Table 2:** Univariate linear mixed effects model using BMI category as a predictor for rocuronium maintenance dose in mg/kg of IBW. All patients should receive similar doses based on IBW, but in this study obese patients received higher doses.

BMI category	Estimate (mg/kg of IBW)	*p* value
Very severely underweight	0.287	0.97
Severely underweight	0.285	0.98
Underweight	0.283	0.83
Normal (reference)	0.286	
Overweight	0.294	0.09
Obese class I	0.293	0.18
Obese class II	0.304	**0.02**
Obese class III	0.346	**<0.0001**

**Table 3:** Univariate linear mixed effects model using provider experience and training level as a predictor for rocuronium maintenance dose in mg/kg of IBW. More inexperienced providers (CA1s and SRNAs) tended to give smaller doses than their more experience colleagues.

Provider training level	Estimate (mg/kg of IBW)	*p* value
CRNA (reference)	0.291	
Anesthesiology fellow	0.315	**0.0002**
CA3	0.312	**0.0005**
CA2	0.309	**0.0005**
CA1	0.269	**0.0002**
SRNA	0.273	**0.008**
